# Biological Cardiac Tissue Effects of High-Energy Heavy Ions – Investigation for Myocardial Ablation

**DOI:** 10.1038/s41598-019-41314-x

**Published:** 2019-03-21

**Authors:** Felicitas Rapp, Palma Simoniello, Julia Wiedemann, Karola Bahrami, Valeria Grünebaum, Svetlana Ktitareva, Marco Durante, P. Lugenbiel, D. Thomas, H. Immo Lehmann, Douglas L. Packer, Christian Graeff, Claudia Fournier

**Affiliations:** 1 GSI Helmholtzzentrum für Schwerionenforschung GmbH, Darmstadt, Germany; 2 Department of Science and Technology, Parthenope University of Naples, Naples, Italy; 3 Technische Universität Darmstadt, Institut für Festkörperphysik, Darmstadt, Germany; 4 Department of Cardiology, University Hospital Heidelberg, Heidelberg, Germany; 5 HCR (Heidelberg Center for Heart Rhythm Disorders), Heidelberg, Germany; 6 DZHK (German Center for Cardiovascular Research), partner site Heidelberg/Mannheim, Heidelberg, Germany; 7 University of Pittsburgh Medical Center, Pittsburgh, PA USA; 8 Mayo Clinic/St. Marys Hospital, Rochester, MN USA

## Abstract

Noninvasive X-ray stereotactic treatment is considered a promising alternative to catheter ablation in patients affected by severe heart arrhythmia. High-energy heavy ions can deliver high radiation doses in small targets with reduced damage to the normal tissue compared to conventional X-rays. For this reason, charged particle therapy, widely used in oncology, can be a powerful tool for radiosurgery in cardiac diseases. We have recently performed a feasibility study in a swine model using high doses of high-energy C-ions to target specific cardiac structures. Interruption of cardiac conduction was observed in some animals. Here we report the biological effects measured in the pig heart tissue of the same animals six months after the treatment. Immunohistological analysis of the target tissue showed (1.) long-lasting vascular damage, i.e. persistent hemorrhage, loss of microvessels, and occurrence of siderophages, (2.) fibrosis and (3.) loss of polarity of targeted cardiomyocytes and wavy fibers with vacuolization. We conclude that the observed physiological changes in heart function are produced by radiation-induced fibrosis and cardiomyocyte functional inactivation. No effects were observed in the normal tissue traversed by the particle beam, suggesting that charged particles have the potential to produce ablation of specific heart targets with minimal side effects.

## Introduction

The establishment of catheter ablation, which uses mostly radiofrequency energy or cryothermal technology, was an important step forward in the treatment of cardiac arrhythmias and has become a widely used and successful method of treatment^1,2^. However, in a number of cases recurrence of arrhythmias occurs^3^. In addition, complications as for instance, an increased stroke risk, bechance^4^. Recently, initial clinical tests of stereotactic X-ray therapy have been successfully performed in patients affected by ventricular tachycardia^5^. These results have triggered a large interest in noninvasive radiotherapy applications in cardiology.

Specific physical characteristics of charged particles permit precise dose delivery including a maximum dose deposition followed by a sharp energy fall-off at the end of the particle trajectory (Bragg peak). This enables a high dose concentration to small target areas in complex anatomical locations. Pencil beam scanning allows high conformal dose distribution in the target^6^. Furthermore, Bragg peak carbon ions demonstrate an enhanced biological effectiveness in terms of cell killing compared to photons^7^, making them a powerful tool for precise irradiation of small targets. For these very reasons, we had proposed that accelerated charged particles could be superior to conventional radiotherapy for treatment of cardiac lesions^8,9^. We have tested this hypothesis first with a Langendorff model^10^ and then in live pigs^11^. The results of the experiment in the porcine model showed slowing and interruption of cardiac impulse propagation in three different targets, i.e. atrioventricular junction (AVJ), left atrial pulmonary vein junction and left ventricular tissue (LV)^11^.

Although our translational study for this treatment modality has elucidated feasibility to use charged particles in a clinical setting, many of the underlying/molecular principles are still unclear. In the present study, we aimed to investigate how the overall damage pattern in the target region is related to the electrophysiological changes. To this end, we analyzed the dose response for fibrosis induction at a microscopic level. Further, we investigated whether changes related to functionality are detectable in cardiomyocytes. In addition, this study sought to identify occurrence of toxicity in myocardium or other tissues within the entrance channel region, e.g. skin, and/ or at unirradiated tissue sites.

## Material and Methods

### Study overview

In total, seventeen pigs (*sus scrofa domestica*) of either sex (weight of 30–35 kg, age ~10 weeks) were randomized to different irradiation target locations and evaluation timepoints (for details, please see the detailed description in our previous report on this study^11^). A baseline study assessing pertinent cardiac parameters was performed for all animals to enable later evaluation of irradiation effects. Endpoints were prospectively selected. All animal procedures were approved by the regional board of the state of Baden-Württemberg, Karlsruhe, Germany (approval number G-7/14) as well as the committees of GSI Helmholtzentrum für Schwerionenforschung GmbH, Darmstadt, Germany (GSI). All animal procedures were carried out in accordance with the ‘German Law for Animal Research’ (Tierschutzgesetz) and with the NIH Guide for the Care and Use of Laboratory Animals as well as the guidelines established by the Mayo Foundation animal care and use committee.

For the detailed investigations presented here, five animals receiving atrioventricular junction (AVJ) irradiation, and one animal receiving sham procedure were analyzed 6 months after irradiation. Below, they are referred to as animal #1, #2 (both 55 Gy), #4 (40 Gy), #7, #8 (both 25 Gy) and # 11 (0 Gy) following the nomenclature of the first publication^11^.

### Irradiation technique

Irradiation was carried out on a fixed horizontal beam line using raster scanning at GSI. In brief, animals were sedated during irradiation, and oxygenation was maintained through intermittent-positive pressure ventilation^11^. Sedated animals were immobilized and both 4D cardiac CTs and irradiation were carried out under enforced breath hold. Two opposing LR (left-right) fields were used and the spread-out Bragg-peak (SOBP) was generated by active energy variation. The exact SOBP energy and width varied depending on the specific animal anatomy. The minimum-maximum 12C-ion energy range for the different animals was between 110 and 260 MeV/n, and the SOPB width ranged from 20 to 50 mm, obtained with iso-energy slices spaced 3 mm and a ripple filter for uniform dose. The mid-SOBP dose-averaged LET ranged 55–75 keV/µm depending on the size of the SOBP and the initial energy. Two opposing field were optimized separately to deliver the target dose to the AVJ while specifically considering doses to trachea, esophagus and aorta^12^.

The target volume (a 5 mm^3^ sphere for AVJ) was isotropically expanded by 5 mm and a range-dependent ITV was calculated on the 4DCT^13^. A slice-by-slice rescanning scheme was applied to counter the interplay effect^14^. Plans were accepted if > 95% of the target dose was delivered to the AVJ in 4D simulation.

### Collection of samples

As described previously^11^, samples from AVJ target (T), entrance channel (EC) and outfield regions (OUT) were collected. Briefly, tissue samples were fixed in 4% PFA at 4 °C over night, dehydrated, incubated in melted paraffin at 58 °C embedded and cut in 5–7 µm section using a microtome. Skin samples of EC and OUT regions were collected and processed similarly. Skin doses were assessed on the outer 3 mm of the body contour (~17 Gy in EC, 0 Gy in OUT). Irradiated samples were compared with corresponding outfield samples of the same animal.

### Histological stainings and analysis

After deparaffinization, sections were stained with Hematoxylin-Eosin (HE), Verhoeff-Van Gieson, Prussian Blue or Masson-Trichrome. Pictures were taken with a light microscope (Olympus BX61) at different magnifications indicated below and further analyzed as described.

For the analysis of hemorrhage, at least 50 pictures per AVJ sample were analyzed in HE-stained sections (40×). The analysis was performed with FIJI (“FIJI is just ImageJ”^15^) by examining stained erythrocytes. To this end, the original RGB images were separated into single color spaces (red, green, and blue channel). Best contrast between erythrocytes was displayed by the green color space, shown in black, while tissue is displayed in gray (Fig. S1). After selection of all black erythrocytes by threshold settings, the percentage of area was calculated and exported to MS Excel or GraphPad Prism (Version 7).

Hemoglobin of erythrocytes is degraded to hemosiderin by macrophages and forms iron- containing precipitates^16^. The reaction of iron-III to ferric ferrocyanide in these siderophages was visualized according to^17,18^, and cell nuclei were counterstained with Nuclear Fast Red. At least 50 pictures per AVJ sample were analyzed (40×). A cell was only counted as a siderophage when the blue stained hemosiderin was located next to a red stained nucleus.

Microvessels (capillaries) were analyzed in Verhoeff-Van Gieson stained sections (100×) with regard to number per field of view and diameters. Only regions of cross-sectioned myocardium were used, and only cross-sectioned capillaries were evaluated to obtain comparable results. Per sample, 40–60 pictures were analyzed using FIJI and the built-in ellipse function to mark microvessels in combination with the ROI setting and measurement of Feret’s diameter.

To give the best tissue overview for analysis, images of hemorrhage and siderophages were taken at randomly distributed locations of sections. For microvessel analysis however, fields of view had to be chosen carefully in order to find suitable myocardial cross-sections. In this process, regions of intense hemorrhage or fibrosis had to be excluded, as cardiac morphology was not discernible. Thus, heavily damaged regions were excluded from this type of analysis.

Fibrosis was visualized using Masson-Trichrome staining. Areas covered by fibrotic collagen fibers were determined semi-quantitatively and staged as not present, weakly, moderately, and strongly present (*, **, *** and **** respectively). Per sample, 5 sections were analyzed by microscopic examination, and fibrotic tissue was evaluated on the total area of each section per field of view. In addition, immunohistochemical staining for α-smooth muscle actin (α-SMA) was performed.

### Immunohistochemistry

Immunohistochemical stainings were performed as described in^19^. Primary antibodies used are anti-Troponin T cardiac isoform (Thermo Scientific); anti-CD45, α-SMA, anti-Cytokeratin 10 and anti-Ki 67 (all from Abcam). For detection of binding of the primary antibody, the Ultra-Sensitive ABC Peroxidase rabbit/mouse IgG staining kit (Thermo Scientific, Waltham, MA, USA) and the ImmPACT VIP-Peroxidase substrate kit (Vector, Burlingame, CA, USA) were used according to the respective manufacturer’s protocols. Nuclei were counterstained with hematoxylin and slides were dehydrated, cleared in xylene, and mounted with Eukitt (Sigma Aldrich, USA).

### Statistics

Graphs and statistics for analysis of hemorrhage, siderophages, and microvessels were created with GraphPad Prism (Version 7).

## Results

### Electrophysiological outcome

Electrophysiological changes were measured by electroanatomical voltage mapping and occurred in the exposed animals in a dose dependent manner^11^. For the AVJ, these are summarized in our previous paper^11^ in Table 1 and 2. In brief, the low dose of 25 Gy did not result in electrophysiological changes (2 animals). In one animal irradiated with 40 Gy, a transient AV block developed (three other animals of this group were excluded). In the group irradiated with the highest dose of 55 Gy, one animal had a complete AV block. One animal was excluded, and in one animal of this group, the lesion was misplaced due to technical reasons; therefore, it did not develop an AV block, but histological changes.

Below, we will describe the biological effects of the irradiation.

### Hemorrhage and Siderophage numbers 6 months after C-ion irradiation

**Figure 1:**
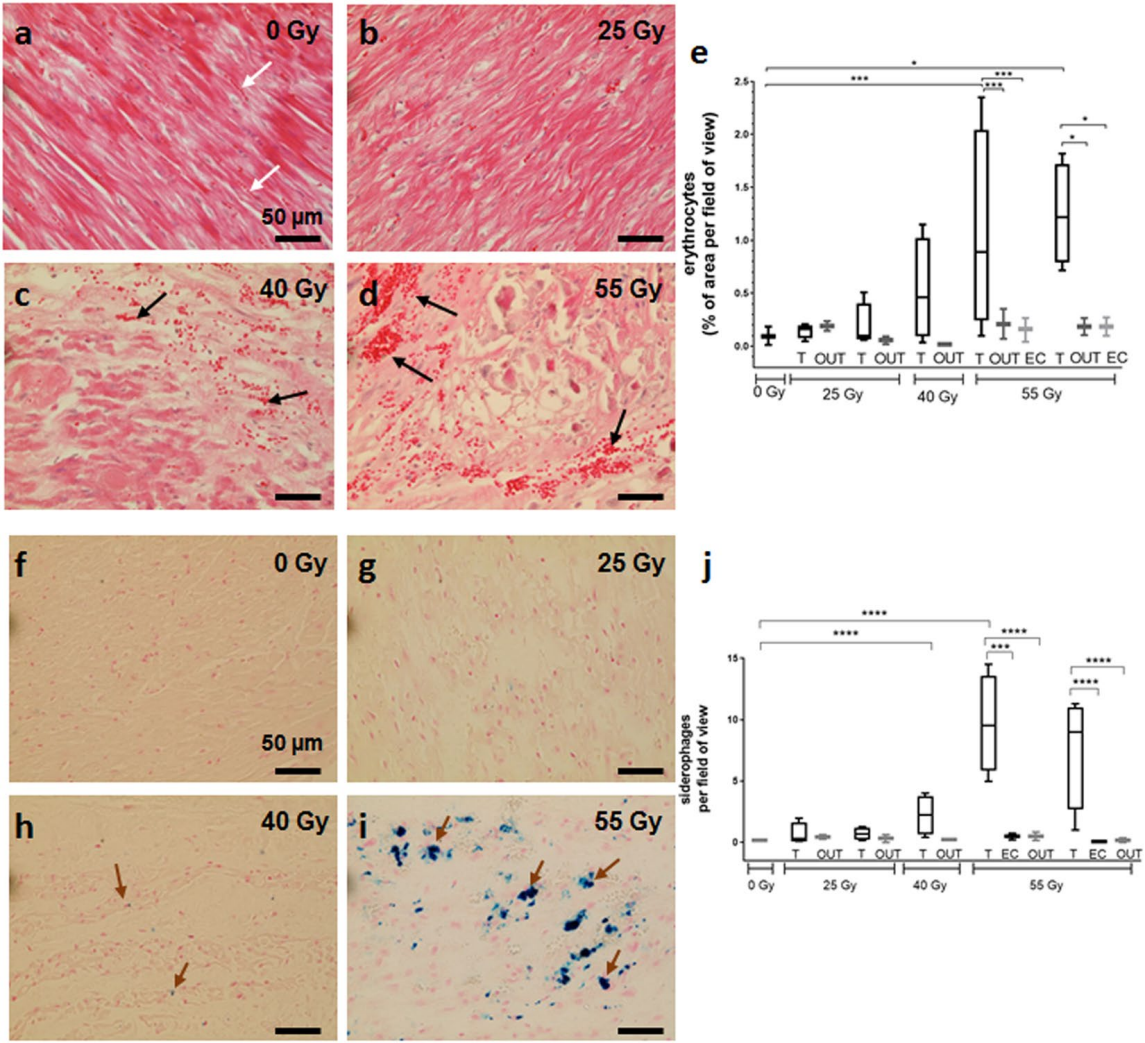
Analysis of bleeding and siderophages in samples of pig hearts six months after targeted C-ion irradiation of the atrioventricular junction (AVJ). Samples from target region (T), entrance channel (EC) and outfield (OUT) were analyzed. Bleeding was quantified in HE stained sections by detection of erythrocytes (**e**). In irradiated AVJ regions, bleeding is persistent even after 6 months (**a**–**d**: 0–55 Gy; black arrows), whereas in samples from EC and OUT regions, only background levels of erythrocytes were detected (white arrows). ***p = 0.0005; *p = 0.0305. Hemosiderin deposits in siderophages were visualized with Prussian Blue staining (**f**–**i**; 0–55 Gy; brown arrows). Siderophages were counted per fields of view. Only after the highest dose of 55 Gy, a significant increase was measured (**j**). In samples from EC and OUT regions, siderophage levels did not differ from sham irradiated control tissues. ****p-value < 0.0001; ***p-value = 0,0001. Tukey Box plot and median (+/−SD). Significance was tested with one-way ANOVA. 0 (N = 1), 25 (N = 2), 40 (N = 1) and 55 Gy (N = 2); n = 3 (0 Gy); n = 4 (t); n = 2 (out); n = 2 (ec); k = 310.

Bleeding was analyzed 6 months after irradiation. In the tissue of the sham irradiated animal, erythrocytes were mainly found in intact capillaries (Fig. 1a, white arrows). This constitutes the physiological background value in myocardium. In target regions, erythrocytes were often found outside of vessels (Fig. 1b–d, black arrows) indicating hemorrhage into the myocardium. For the sham irradiated animal (0 Gy), background levels of erythrocytes present in analyzed pictures were less than 0.1% of the area, whereas in target samples of the irradiated pigs, the area covered by erythrocytes increased to ~0.2% (25 Gy), 0.5% (40 Gy) and ~1% (55 Gy), respectively (Fig. 1e). It should be pointed out that the variation of bleeding in pictures is very high, indicating region specific differences and resulting in the depicted high interquartile ranges for all irradiated tissues. In samples that were taken from OUT regions of irradiated pigs, the amount of bleeding ranged from 0.02 to 0.19%. In EC regions, which were exposed to ~17 Gy, values for the erythrocyte covered areas were not significantly different from levels found in sham irradiated animals (tested for 55 Gy).

In response to bleeding, macrophages migrate into tissue and dispose of erythrocytes. We visualized siderophages using Prussian Blue staining (Fig. 1f–i, arrows). The number of siderophages was counted in microscopic pictures. After sham irradiation, the number of siderophages is low (below 1 siderophage/field of view), but increases with dose (below 1 for 25 Gy and up to ~10 for 55 Gy; Fig. 1j). Siderophage numbers are significantly higher in samples irradiated with 40 and 55 Gy compared to sham irradiation as well as to EC or OUT regions of the same animals. The EC samples for 25 and 40 Gy were not further analyzed because no effects were detectable even in EC samples irradiated with 55 Gy.

Taken together, bleeding in the target regions was still detectable 6 months after irradiation. EC and OUT regions were not significantly different from sham irradiation levels. In addition, siderophages indicating clearance of erythrocytes in tissue from previous bleeding were observed in a dose-dependent matter.

### Microvascular damage

**Figure 2:**
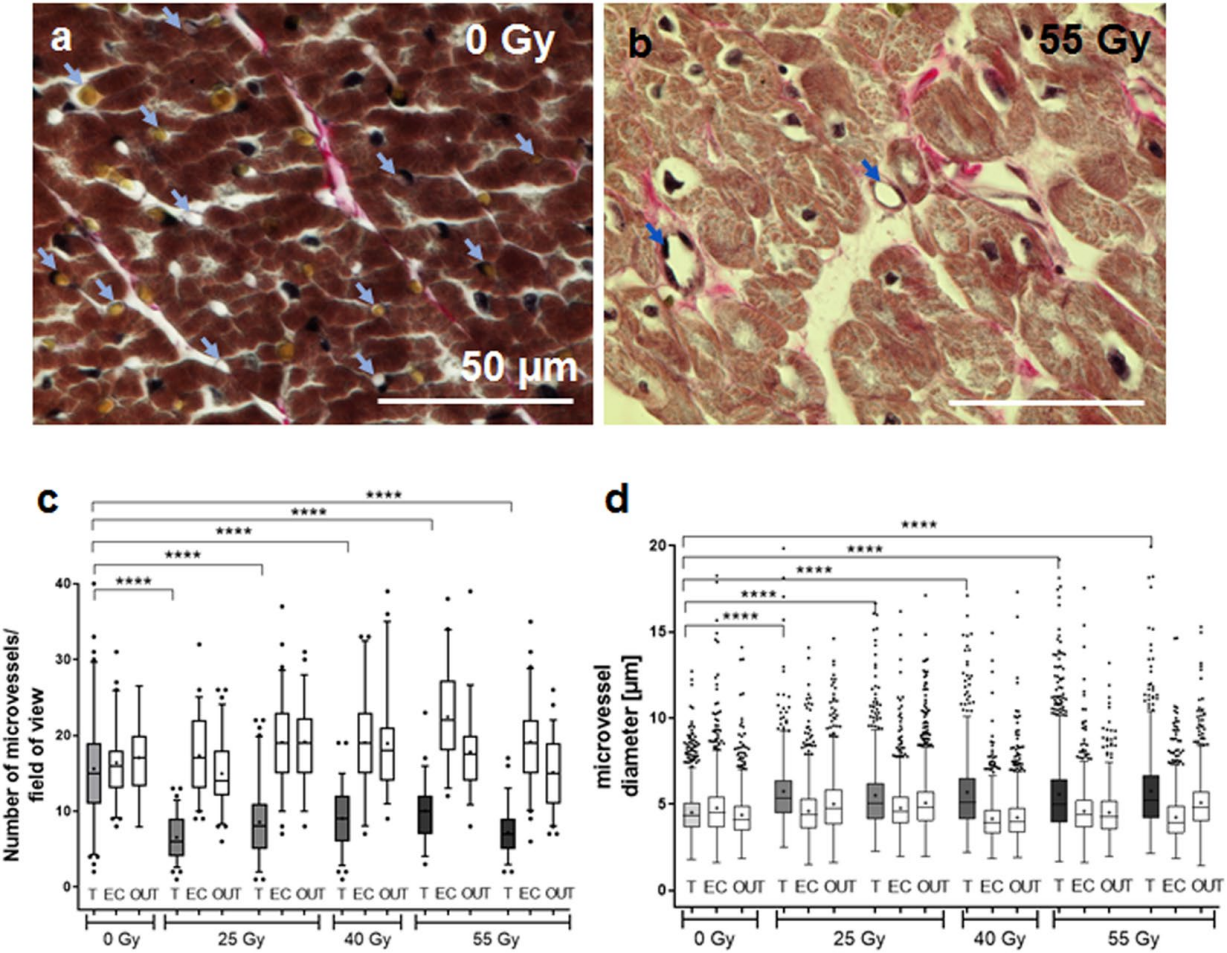
Analysis of microvessels in samples of pig hearts six months after targeted C-ion irradiation of the atrioventricular junction (AVJ). Vessels numbers (capillaries) and diameters per fields of view were measured in cross sections from target region (T) as well as entrance channel (EC) and outfield (OUT) regions. Representative cross sections are shown in (**a**) (0 Gy) and (**b**) (55 Gy); capillaries are indicated by arrows. The total number of microvessels is significantly reduced six months after irradiation in target regions (**c**), but not in ec or out regions. The diameter of microvessels is increased in target regions (**d**), whereas ec and out regions are not changed compared to sham irradiated control levels. Tukey Box plot and median (+/−SD; − = median, + = mean); One-way ANOVA. ****p < 0.0001.

To analyze if microvasculature in the irradiated AVJ tissue is damaged, we used Verhoeff-Van-Gieson stained cross-sections of myocardium to identify capillaries (Fig. 2a,b, arrows). The number of capillaries per field of view and their respective diameters were measured and counted. As shown in Fig. 2c, after treatment, the number of microvessels decreased significantly in irradiated AVJ samples compared to sham irradiation and to samples from EC or OUT regions of the same animal. In parallel to loss of microvasculature, capillaries in irradiated regions (AVJ, T) were slightly larger in diameter (Fig. 2d) compared to sham irradiated tissue or unirradiated regions from the same animal. Although increase in diameter in the targeted regions was not pronounced when referring to the mean values, it is obvious that in some parts of the tissue the difference is large. Please note that this method of analysis could only be carried out in parts of tissue where microvessels still could be identified. Heavily damaged areas, i.e. with complete scar, were automatically excluded for technical reasons.

In summary, we observed radiation induced local damage and tissue reactions which are still ongoing 6 months after the treatment, indicating a persistent effect on the myocardium. In contrast, the regions outside the target (AVJ) were not affected as shown by our analyses (Figs 1e,j and 2c,d).

### Late Cardiac Tissue Reactions- Inflammation and Fibrosis

**Figure 3:**
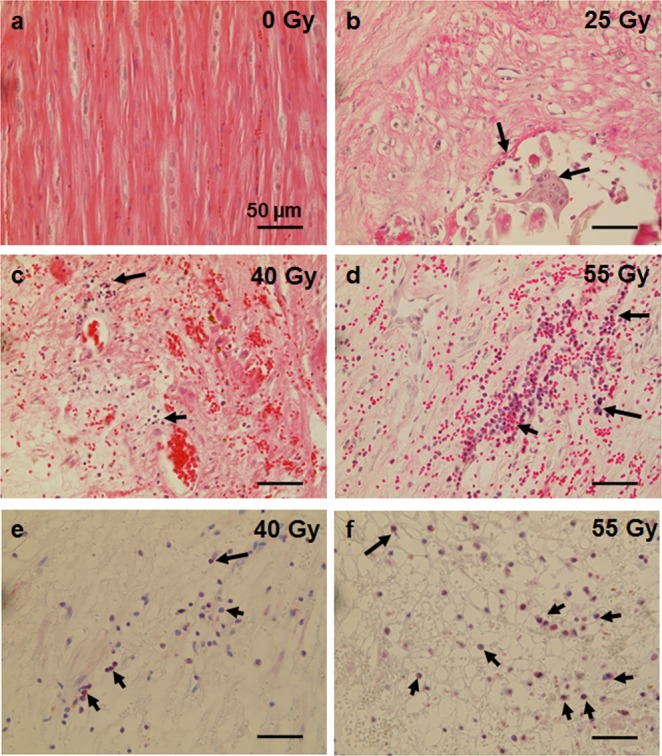
Inflammation in samples of pig hearts six months after targeted C-ion irradiation of the atrioventricular junction (AVJ). Samples from target region (T) as well as entrance channel (EC) and outfield (OUT) regions were HE-stained and examples are shown in (**a**–**d)** (0–55 Gy). Arrows indicate immune cell infiltrations. For a more specific identification, sections were stained with an CD45-antibody to visualize T-cells ((**e**) 40 Gy and (**f**) 55 Gy; pink: CD45 positive cells; examples shown by arrows), which matches well with the HE staining.

In irradiated target tissues, elevated levels of inflammatory cells were still present 6 months after C- ion irradiation as shown in HE- stained sections (Fig. 3a–d, black arrows), most likely due to immune cell infiltration in irradiated tissue areas. In addition, immunohistochemical stainings were used to identify activated T-cells (CD45^+^, Fig. 3e,f), demonstrating that inflammatory reactions are mostly present in tissues irradiated with higher doses, and in regions where tissue is visibly damaged. The injuries or tissue alterations were characterized in more detail as follows.

**Figure 4:**
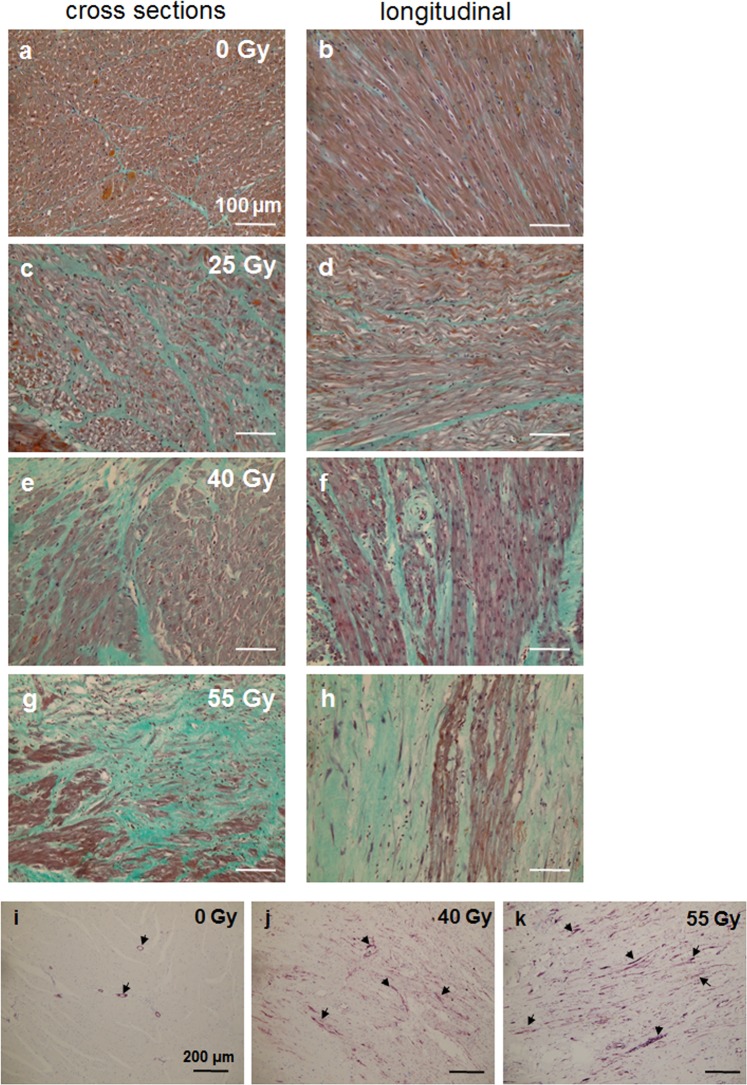
Fibrosis induction in samples of pig hearts six months after targeted C-ion irradiation of the atrioventricular junction (AVJ). The AVJ was irradiated with 25, 40 or 55 Gy. Examples of Masson- Goldner Trichrome stained sections are shown in (**a**–**h**) (0–55 Gy); left panel shows cross sections, right panel shows longitudinal sections. Blue/black- nuclei; red/brown- cytoplasm; red/pink- erythrocytes; green- collagen. In samples from sham irradiated animals, myocardium is intact as indicated by wide areas covered with brown cardiomyocytes and collagen found only in connective tissue in the septa (**a,b**). In irradiated AVJ samples, interstitial collagen deposition is aggravated (**c–h**), indicated by an increased area stained in green and less intact cardiomyocytes. To distinguish between healthy/normal connective tissue and pathological collagen deposited by myofibroblasts, sections were immunostained for α-smooth muscle actin (α-SMA). In sham-irradiated tissue, only the perivascular myofibroblasts are positive (**i**), whereas in irradiated tissue, myofibroblasts between cardiomyocytes are visible ((**j)**, 40 Gy and (**k**), 55 Gy; α-SMA: pink; examples are marked by arrows).

As a reaction to radiation-induced tissue injuries like vascular rupture, bleeding or cardiomyocyte damage, the development of fibrosis is a well-known effect^20,21^. When using irradiation as a tool for cardiac ablation, this is even desired to block signal conduction. We visualized fibrosis with Masson-Trichrome staining, where collagen fibers are stained green, cytoplasm of intact cells is stained brownish and nuclei blue/black (Fig. 4a–h). The physiological occurrence of collagen, e.g. in connective tissue surrounding bigger vessels or between segments of myocardium, is depicted in Fig. 4a,b (for a cross section and a longitudinal section, respectively) and constitutes the tissue specific physiological level. In contrast, the fibrotic reaction, which is an increase of interstitial collagen deposition, can be seen with increasing dose (25 Gy in C and D, 40 Gy in E and F, 55 Gy in G and H). Collagen from connective tissue in healthy myocardium can be discriminated from newly deposited collagen during a scar formation by the presence of α-smooth muscle actin (α-SMA^22^). After damage to the myocardium, α-SMA is expressed along with collagen by reactive myofibroblasts, which participate in wound healing and scar formation. In Fig. 4i, sham irradiated tissue is shown, where α-SMA only occurs around vessels in smooth muscle cells forming the tunica media (immunohistochemical staining, α-SMA shown in pink, black arrows). In irradiated tissue (Fig. 4j,k), an increase in α-SMA expression is also visible interstitially.

**Figure 5:**
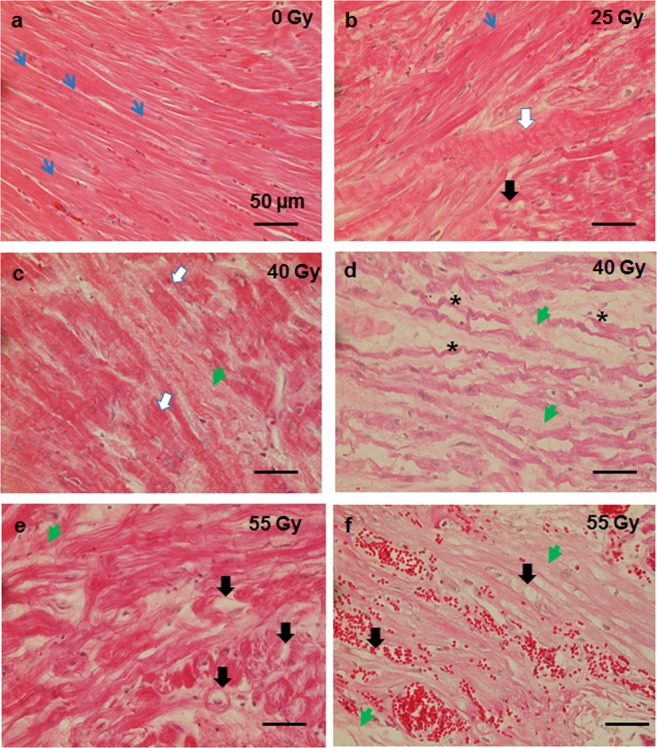
Structural changes in cardiomyocytes from samples of pig hearts six months after targeted C-ion irradiation of the atrioventricular junction (AVJ). Analysis was performed in HE-stained longitudinal sections of myocardium. Examples are shown in (**a–f**). In A, sham irradiated myocardium with intact cardiomyocytes is shown. Regular striation is indicated by blue arrows. With increasing dose cardiomyocyte damage is more pronounced ((**b**) 25 Gy, (**c** and **d**) 40 Gy, (**e** and **f**) 55 Gy). After 25 Gy, regions with normal cardiomyocytes are present (blue arrows), but in parallel, changes like wavy fibers (white arrow), vacuolization (black arrows) or collagen deposition (green arrowheads) occur. After 40 and 55 Gy, damage to cardiomyocytes is more severe, as shown by larger amounts of vacuolization, loss of striation (*), loss of polarity, bleeding and more collagen deposition. (**c**,**d**) as well as (**e**,**f**) are from different regions of one animal, respectively.

Fibrosis can be a cause for disrupted electrophysiological signaling, but also cardiomyocytes are changed directly by irradiation^23^. Although we did not find indications for the occurrence of apoptosis at this late time point (6 months; Fig. S3 and^11^), cardiomyocytes in the target tissue (AVJ) displayed changes in morphology (Fig. 5). In sham irradiated tissue, cardiomyocytes are tightly connected, show the normal striation and a regular shape of nuclei (Fig. 5a, blue arrows). In irradiated AVJ tissues, cardiomyocytes exhibit a dose dependent increase in the occurrence of wavy fibers, vacuolization, loss of striation and loss of polarity. After irradiation with 25 Gy of C- ion irradiation, the myocardium in the target area does not show alterations in wide parts (Fig. 5b, blue arrow), but some wavy fibers were present (white arrows), along with single vacuolated cardiomyocytes (black arrow). After exposure to 40 Gy, interstitial collagen (Fig. 5c,d, green arrows) as well as wavy fibers (white arrows), and loss of striation and polarity (*) were detected. Most pronounced changes were present in tissue irradiated with 55 Gy (Fig. 5e,f). In Fig. 5f, bleeding was also visible as can be inferred by many erythrocytes stained in bright pink.

### Irradiation Induced Cardiomyocyte Alterations

**Figure 6:**
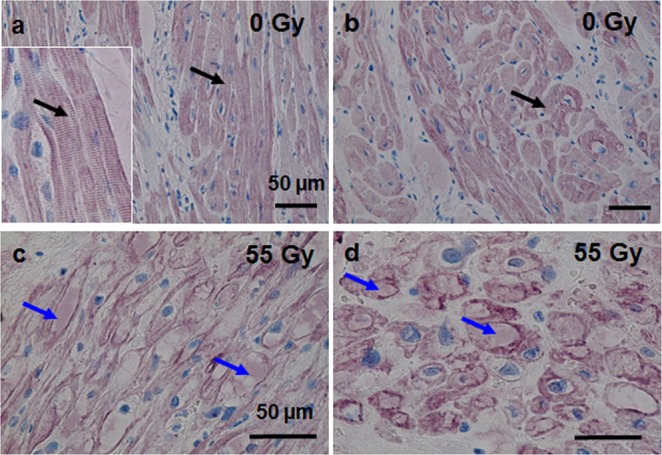
Cardiac Troponin T distribution in myocardium of pigs six months after targeted C-ion irradiation of the atrioventricular junction (AVJ). Analysis was performed in immunohistochemical stainings (cardiac Troponin T (cTropT, pink/ violet). (**a** and **b**) sham irradiated tissue (0 Gy, (**a**) longitudinal section, (**b**) cross section). In the longitudinal section, the typical striation of cardiomyocytes is visible (black arrows; insert). In irradiated tissue (55 Gy, (**c**) longitudinal, (**d**) cross section), localization of cTropT is partly lost in myofilaments or occurs in the cytoplasm (blue arrows).

To further characterize irradiation-induced damage in cardiomyocytes, we applied immunohistochemical stainings for a typical cardiomyocyte protein, cardiac troponin T (cTropT). cTropT is a calcium binding protein enabling cardiomyocyte contractions^24^. It is typically bound to the myofibrils of cardiomyocytes, and some molecules are present in the cytoplasm^25^. For the analysis of the marker proteins, the orientation of the tissue has to be considered. In longitudinal sections of myocardium, the staining pattern will look different from that in cross-sections. Therefore, both orientations are shown. cTropT staining in sham irradiated tissue from AVJ is located in intact sarcomeres throughout the cytoplasm (Fig. 6a; black arrows; cTropT in purple, nuclei in blue). In the insert, the structure of a striated cardiomyocyte is illustrated in more detail. In cross-sections of sham irradiated AVJ tissue, the striation is not visible, but also here the distribution of cTropT is spread over the cytoplasm and around the nuclei (Fig. 6b). In longitudinal sections of the irradiated AVJ tissue (55 Gy, 6 months), striation is lost (Fig. 6c, blue arrows). In cross-sections, cTropT is mostly detected at the edges of the cytoplasm of the cardiomyocytes (Fig. 6d), most likely a consequence of vacuolization in the cells.

In Table 1, a semi-quantitative analysis of fibrosis and the expression of cardiac Troponin T is shown. Microscopic evaluation per fields of view shows an increase of fibrosis with dose, but also an inter-individual difference between animals of the same irradiation group. For example, animals #7 and #8 both received 25 Gy, but animal 7 developed more fibrosis than animal 8. This is in accordance with the macroscopic lesions reported in our previous results^11^. Carbon ion irradiation results in changes in the cellular distribution of cTropT and loss of its expression in the striation.

**Table 1:** Semi-quantitative analysis of fibrosis induction and vacuolization in the AVJ.

Animal #	Dose [Gy]	Fibrosis (collagen)	Vacuolization and loss of Cardiac Troponin T
1^++^	55	****	****
2	55	***	***
4^+^	40	***	****
7	25	**	**
8	25	*	**
11	0	*	*

Samples were evaluated microscopically. Animal #1 (++) had persistent AV block, animal #4 (+) had transient AV block). In animal #2, the lesion was misplaced due to technical issues.

For fibrosis analysis, the area covered by pathologically deposited collagen was noted. *No fibrosis in the total area; **less of 50% fibrosis in the total area; ***more than 50% of fibrosis in the total area; ****~80% of fibrosis.

For quantification of vacuolization and loss of cardiac proteins, cardiac Troponin T staining was used. *Regular distribution; **>50% of cardiomyocyte area covered with troponin; ***<50% of cardiomyocyte area covered with troponin; ****almost no regular distribution.

### Absence of Irradiation Toxicity in Non-targeted Skin Tissue

**Figure 7:**
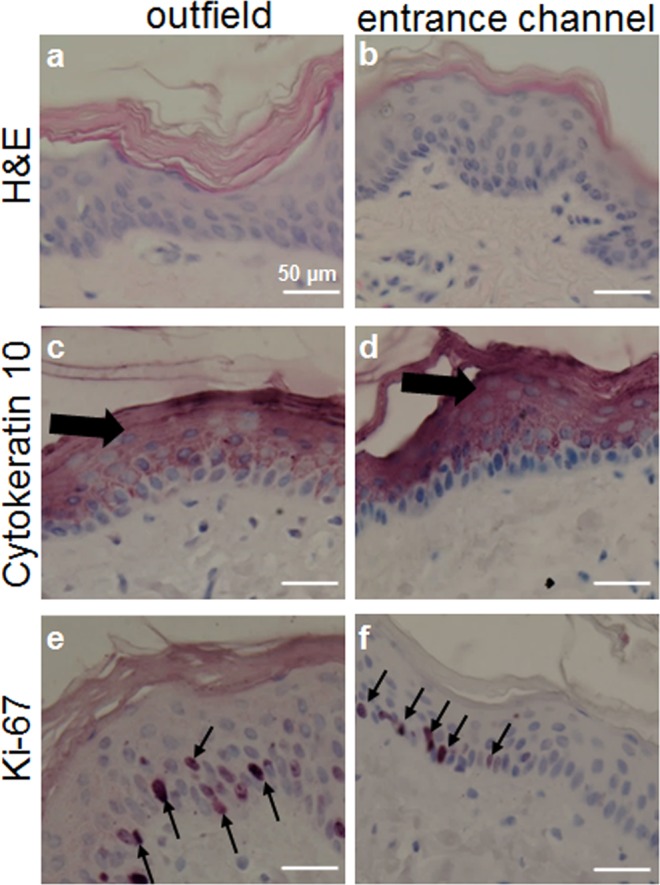
Analysis of skin in the entrance channel during C- ion irradiation of the atrioventricular junction (AVJ) in the heart. Sections were stained with HE (**a,b**), or immunostained with an antibody against Cytokeratin 10 (keratinocytes, (**c,d**) pink, thick arrows), or Ki67 (proliferating cells, (**e,f**) thin arrows). No differences between irradiated skin in EC (calculated dose ~17 Gy) and in OUT samples (0 Gy) were detectable, indicating the absence of side effects.

In order to detect possible toxicity of C- ion irradiation to the AVJ of the heart, we also analyzed skin samples from EC and OUT regions of (sham-) irradiated pigs. HE stainings (Fig. 7a,b) as well as antibody stainings for differentiating keratinocytes (Fig. 7c,d) or proliferating cells (Fig. 7e,f) indicated a well-preserved histology/structure as well as no changes in differentiation or proliferation. This is in accordance with our previous findings of macroscopic analysis of the skin^11^.

## Discussion

For the treatment of arrhythmias and other cardiac disorders with ionizing irradiation, photons (mostly in combination with Stereotactic Body Radiation Therapy (SBRT) or CyberKnife technique) have been used for cardiac ablation^26^. For the use of carbon ions in producing cardiac lesions leading to arrhythmia elimination, we conducted the first explorative study, which we recently published^10,11^. Some experimental data using photon irradiation in swine models^20,27^ showed findings comparable to ours regarding block induction and fibrotic scar formation. In other studies, Carbon ions were used in a rabbit^28,29^ or dog model^30^ after myocardial infarction (MI). Here, the goal was to improve the conductivity of damaged heart tissue and not to interrupt conductivity in normal tissue as in our study. Therefore, total heart irradiation was used instead of targeting cardiac substructures like in our study, and a lower dose of 15 Gy was applied. With this setting, radiation-induced fibrosis was not reported (only in MI regions). Recently, first clinical observations in humans^5,26,31^ were published for photon-induced ablation, underlining the clinical feasibility of noninvasive ablation alternatives.

However, the cellular and molecular mechanisms remain to be fully elucidated. This accounts even more for carbon ions, because the ablative treatment using carbon ions is specific in terms of dose, dose distribution in the tissue and targeted volume, as well as biological effectiveness. As a consequence, the biological processes underlying the electrophysiological changes are not necessarily the same as for photons. Therefore, we present in this study a more detailed investigation of the target and the surrounding tissue located in the entrance channel of the carbon ion beam, which is an essential part of the assessment of efficacy and risk for side effects of this type of radiation treatment.

Although pigs were sacrificed as late as 6 months after treatment, we still observed persistent hemorrhage in the AVJ, indicating massive vascular damage with erythrocyte extravasation which was more pronounced with increasing dose (Figs 1 and 2). However, we did not detect cleaved Caspase-3, a marker for apoptosis, in the overall protein analysis (Fig. S3) at this late time point. This is in line with our results for LV target^11^ where cleaved Caspase-3 was detected in LV samples after three, but not after 6 months, indicating that cell death induction is completed during this time or that Caspase-3 independent mechanisms of cell death are dominant at later timepoints.

After irradiation, in particular the small vessels -capillaries- are prone to rupture due to endothelial damage. This leads to “leakage” of the vessels and release of erythrocytes and other blood components into surrounding tissue^32–34^. With regard to the late time point of investigation, it is obvious that bleeding and vascular damage at that time was not induced directly by irradiation, but by secondary effects, i.e. angiogenic signaling evoked by tissue damage, leading to the formation of unstable vessels, which was discussed as a result of inhibited proliferation of endothelial cells^35,36^. It can be assumed that in the same tissue, leaky or destroyed vessels have led to the observed activity of hemoglobin phagocytic macrophages, so-called siderophages (Fig. 1j), showing that bleeding has occurred also earlier after exposure. Similar effects were found in Left Ventricle (LV) target tissues (Fig. S2), where the amount of bleeding and siderophages was still increased after six months, but not in OUT or EC samples. In line with this, we can demonstrate a loss of micro-vessels, probably reducing the oxygen and nutrition supply of the tissue (Fig. 2c).

We measured a slight increase of the diameter of capillaries in the target region (Fig. 2d), but it has to be kept in mind that heavier damaged regions of tissue were excluded from this analysis for technical reasons. In regions where tissue was more severely altered, capillaries could be even more affected. Thus, this analysis method could result in an understatement of the effect in our data and therefore in a concealment of a possible dose dependency as it was demonstrated for bleeding and presence of siderophages. The capillary dilation is most likely a sign of vascular remodeling related to ongoing inflammation^37,38^, going along with transmigration of cytokines and immune cells from the blood stream into inflamed tissue. In line with this idea, we detected an accumulation of immune cells (CD45+) in the target region (Fig. 3), most likely representing the tissue response on long lasting vascular damage and tissue injury due to atrophy and low oxygen supply. This was also reported by others^33,39,40^ and is known to lead ultimately to activated myofibroblasts and fibrosis^41,42^.

A semi-quantitative analysis of the fibrotic area in the target tissues revealed an increase with dose (Fig. 4). In stitched overview pictures of whole sections of targeted AVJ tissue, the increase of interstitial collagen deposition becomes obvious (Fig. S5). In addition, necrotic areas, bleeding or presumably calcified regions can be distinguished from areas with less damage (Fig. S5, arrows). Notably, extent and spread of the fibrotic areas in histological analyses was not the same in the two animals irradiated with the highest dose. Still, it was corresponding to the macroscopically visible lesion, which was misplaced in one animal most likely due to technical issues. A causal relationship of fibrosis and conductivity has been demonstrated, but whether dose uncertainty or other issues account for the less extended occurrence of fibrosis and the absence of electrophysiological changes in one animal has to be further investigated in a higher number of animals.

The observed injury of target tissue, i.e. vascular damage, subsequent hemorrhage, inflammation and fibrosis, are known effects following radiation exposure of the heart in patients^43–46^ and experimental models^33,47–49^. However, most of the respective studies are performed for whole body or whole/partial heart exposure, which is a completely different situation compared to the scenario of targeting a small volume with a high dose when using C- ions for cardiac ablation.

In addition to vascular damage, inflammation and fibrosis, we observed that cardiomyocytes are still present in the non-fibrotic areas of the target tissue, indicating yet incomplete formation of a fibrotic scar. We detected changes in the cellular structure of cardiomyocytes related to their functionality and similar to those occurring after myocardial infarction^50^. The myocardium of control animals is characterized by regular striation and layers of cells, a prerequisite for polarity and conductivity. In contrast, in the targeted tissue, loss of striation and wavy cardiomyocyte fibers, which are considered to be induced by hydrostatic pressure from edema^51^ were detected (Fig. 5). This morphological change is accompanied by a loss of polarity, which in turn elicits impaired conductivity, although this was not reflected in altered expression level of connexin 43, a structural protein of gap junctions as present in cardiomyocytes (Fig. S4). In addition, vacuolization of the cytoplasm was observed (Fig. 5), indicating cell death and probably followed by phagocytosis of the damaged cardiomyocytes, or also autophagy^52,53^. This so-called myocytolysis is a cardiac lesion, described as being associated with ischemic heart disease and other cardiac lesions where cardiomyocytes die^54,55^.

Of note, no tissue changes were observed in the entrance channel and outfield areas of cardiac (Figs 1 and 2) and skin tissue (Fig. 7). Thus, based on our results, there are no indications for toxicity as they are described elsewhere^44^. This is a very important result of our study, as the risk for cardiovascular disease is considered to be elevated after radiation exposure and the heart has been found to be more sensitive to radiation than assumed in the past^48^. In this context, an advantage of carbon ions is the volume conformity of irradiation. Our results reflect this by the complete absence of effects in the entrance channel (~17 Gy); exposed to doses apparently below the threshold for the massive changes observed in the target area (>25 Gy). In contrast, when photons are used for cardiac ablation, apoptosis of cardiomyocytes outside the target tissue is suspected^27^, albeit not in all studies with a comparable experimental design^20^.

A second advantage of carbon ions is the high dose which can be delivered to the target area. High target doses are discussed as potentially advantageous for cardiac ablation, since the first patients have been treated with single doses of 25 Gy, and one of them had a recurrence^26^. Based on our results, the tissue and cellular effects achieved with carbon ions are increased in a dose dependent manner. It should be noted though that the delivery of scanned particle therapy to moving targets is highly complex^14^. In spite of careful 4D-planning and efforts to suppress target motion^56,57^, the uncertainty in the dose distribution remains high and likely explains the heterogeneous outcome in this study. Nevertheless, this problem also is present with other radiation techniques.

An important inducer of interruption of conductivity is fibrosis, occurring upon carbon ions used for cardiac ablation as well as for photons^20,27^ and electron exposure^58^. However, the concomitant electrophysiological changes occurred earlier after photon compared to carbon ion exposure (photons: 11 weeks, C-ions: 17 weeks).

In the frame of our explorative study, the reason for differences in damage induction cannot be fully explained. Up to now, we cannot exclude that the smaller target volume and the relative sharp fall-off of the target dose in the major part of the surrounding tissue plays a role, in that a rim of exposed tissue around the target “helps” to accelerate scar formation and to interrupt conductivity. This idea is supported by observations made with micro-irradiation of very small target volumes, where in contrast to known effects elicited by irradiation of larger volumes, the surrounding tissue shows very little impact of exposure^59,60^.

However, for C- ions the tissue remodeling which was still ongoing after 6 months with respect to blood vessel integrity, inflammation and cardiomyocyte organization, has not been reported for the target area after photon exposure. Interestingly, for electrons these changes are reported, but seem to be milder than for carbon ions^58^. In the case of carbon ions, persistent tissue remodeling indicates that the formation of a dense fibrotic scar has not been completed yet but is ongoing.

This indicates a relation to the severity of induced damage, i.e. cell death, which increases with the ionizing density of the radiation quality^7^. It remains to be clarified in further studies, whether this bears a disadvantage for the treatment or even an advantage, for example a more sustainable interruption of conductivity because of the more pronounced tissue injury. In a next step, we propose a study with an appropriate number of animals under improved technical conditions to demonstrate the reproducibility of our findings as well as the safety of the procedure. Together with the findings from first case studies with human patients, our results demonstrate the applicability of Carbon ion irradiation as a noninvasive, long-lasting alternative to standard cardiac ablation techniques.

## Supplementary information

Supplementary Dataset 1
