# Supplementary material for: Biological Cardiac Tissue Effects of High-Energy Heavy Ions – Investigation for Myocardial Ablation
Source: Sci Rep. 2019 Mar 21;9:5000. doi: 10.1038/s41598-019-41314-x (PMC6428839; doi:10.1038/s41598-019-41314-x)
Supplement: Supplementary file 1 — Supplementary Dataset 1 [file 41598_2019_41314_MOESM1_ESM.pdf]

Biological Cardiac Tissue Effects of High-Energy Heavy Ions – Investigation for Myocardial Ablation

Felicitas Rapp[1¤], Palma Simoniello[2¤], Julia Wiedemann[1], Karola Bahrami[1], Valeria Grünebaum[1], Svetlana Ktitareva[1], Marco Durante[1], P. Lugenbiel[3,4], D. Thomas [3,4,5], H. Immo Lehmann[6,7], Douglas L. Packer[5], Christian Graeff [1], Claudia Fournier[1]*

[1] GSI Helmholtzzentrum für Schwerionenforschung GmbH, Darmstadt, Germany

[2] Department of Science and Technology, Parthenope University of Naples, Naples, Italy

[3] Department of Cardiology, University Hospital Heidelberg, Heidelberg, Germany

[4] HCR (Heidelberg Center for Heart Rhythm Disorders), Heidelberg, Germany

[5] DZHK (German Center for Cardiovascular Research), partner site Heidelberg/Mannheim, Heidelberg, Germany

[6] University of Pittsburgh Medical Center, Pittsburgh, PA, USA

[7] Mayo Clinic/St. Marys Hospital, Rochester, MN, USA

[¤]equal contribution

*corresponding author (c.fournier@gsi.de)

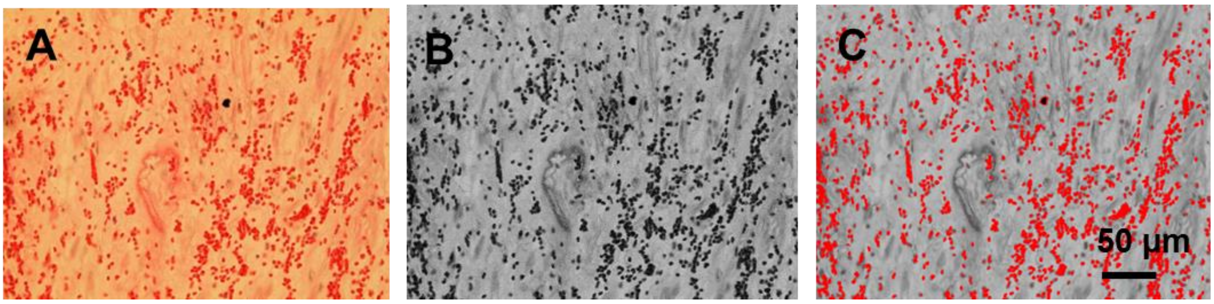

Fig. S1: Analysis of bleeding in HE-stained pictures with ImageJ. The area covered by erythrocytes per field of view was measured. The original image (A) was separated into single color spaces (RGB) by FIJI. In the green channel, contrast between erythrocytes and tissue was most pronounced (B). By threshold setting, erythrocytes (in black) were selected (C, in red) and percentage of area was measured.

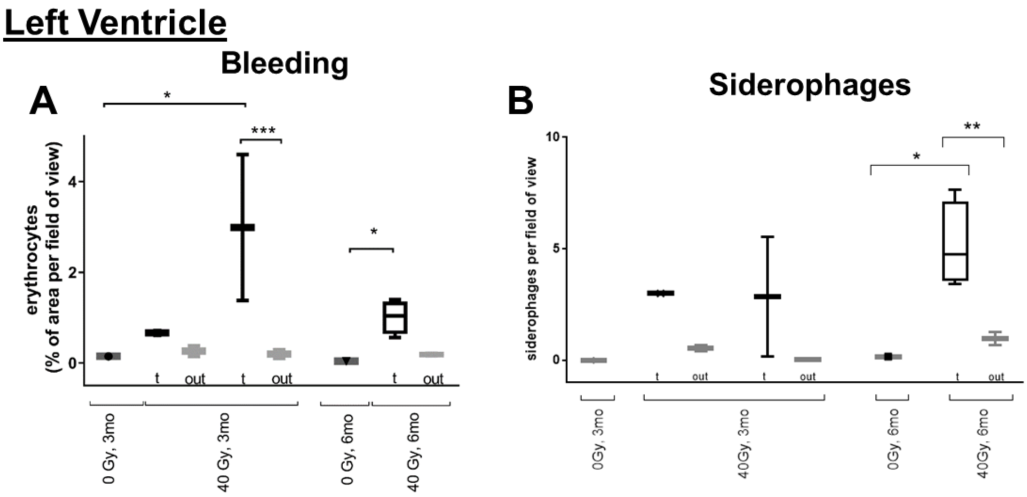

Fig. S2: Analysis of bleeding and siderophages in samples of pig hearts three and six months after targeted C-ion irradiation of the left ventricle (LV). The LV was irradiated with 0 or 40 Gy and samples from target region (t) as well as outfield (out) were analyzed. Bleeding was quantified according to Fig. S1. In irradiated LV regions, bleeding is present after 3 and 6 months, whereas in samples from sham irradiated and outfield regions, only background levels of erythrocytes were detected (A). *** $p < 0.0002$; * $p \leq 0.02$. Siderophages were visualized with Prussian Blue staining and counted per fields of view (B). Similar to fresh bleeding detected by erythrocyte measurements, siderophage numbers are increased after irradiation with 40 Gy at 3 and 6 months in the target regions. In samples from outfield regions, siderophage levels did not differ from sham irradiated control tissues. * $p = 0.039$; * * $p = 0.0125$. Tukey Box plot and median (+/- SD). Significance was tested with one-way ANOVA.

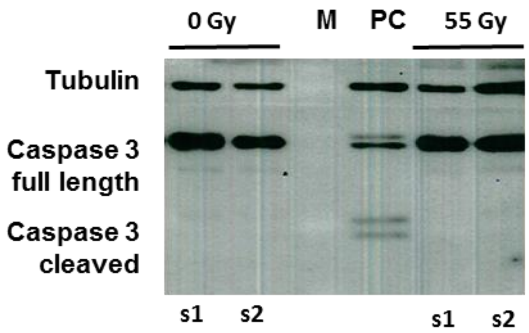

Fig. S3: Analysis of apoptosis in the atrioventricular junction (AVJ) ablation of pig hearts 6 months after targeted irradiation with 55 Gy of Carbon ions. Proteins were extracted from different pieces (s1 and s2) of the irradiated target region and Western Blot analysis was performed. Cleaved caspase-3, an apoptosis marker, was not detected in the AVJ six months after 55 Gy carbon irradiation, but in positive controls of irradiated HaCaT cells. PC: positive control HaCaT (Lysates of HaCaT cells five days after irradiation with 10 Gy of X-ray), M= marker.

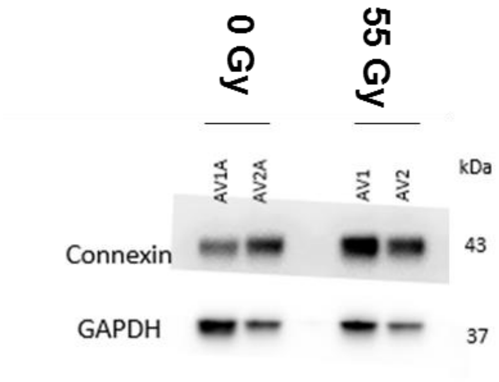

Figure S4: Analysis of gap junctions in the atrioventricular junction (AVJ) in pig hearts 6 months after targeted C ion irradiation (55 Gy). Proteins were extracted from different pieces (s1 and s2) of the irradiated target region and Western Blot analysis was performed. Immunodetection analysis was performed for Connexin (43 kDa) and GAPDH (37 kDa) as housekeeping protein. Samples were normalized and 7 µg of total protein were loaded in each well; N=1 n=1.

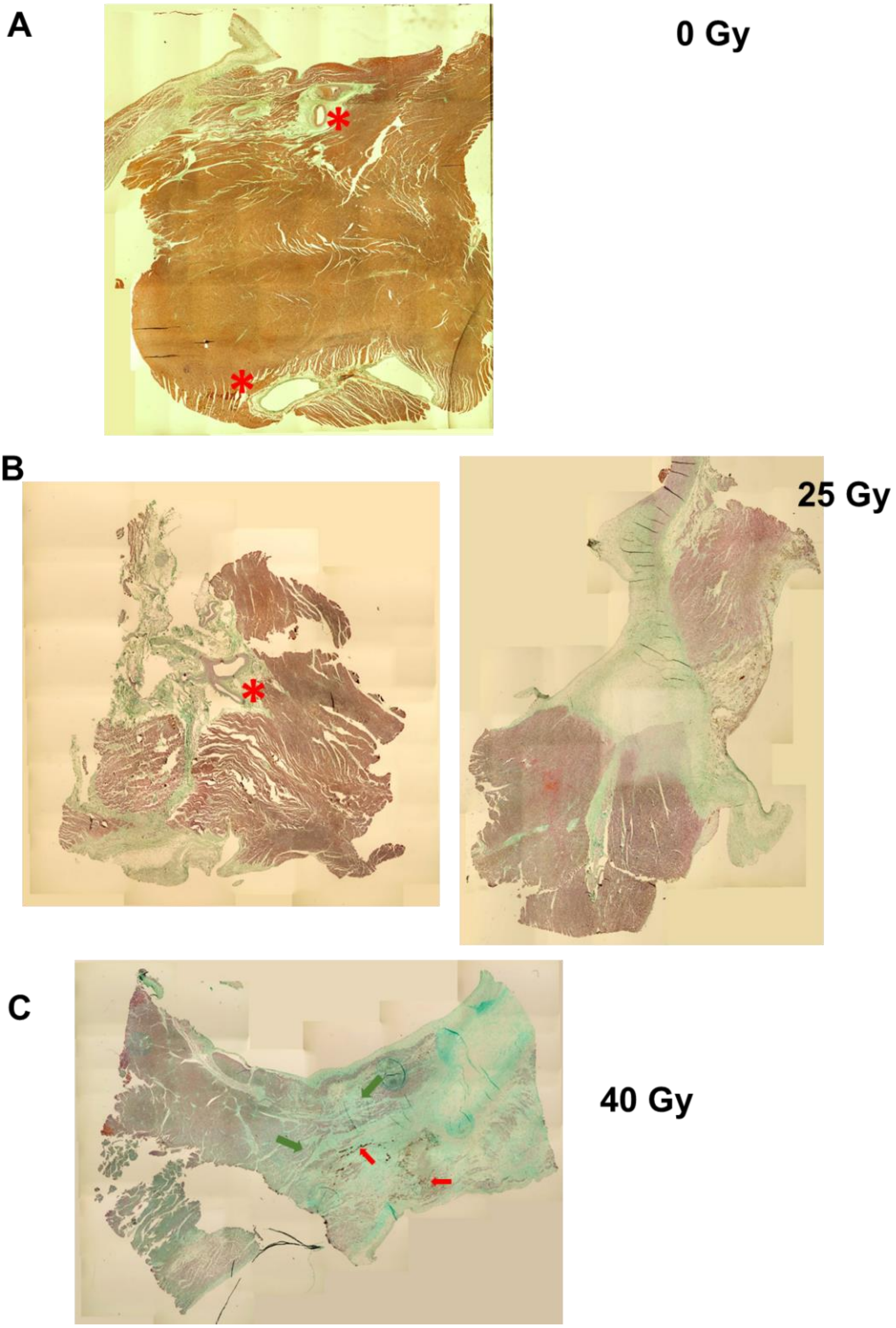
A
0 Gy
B
25 Gy
C
40 Gy

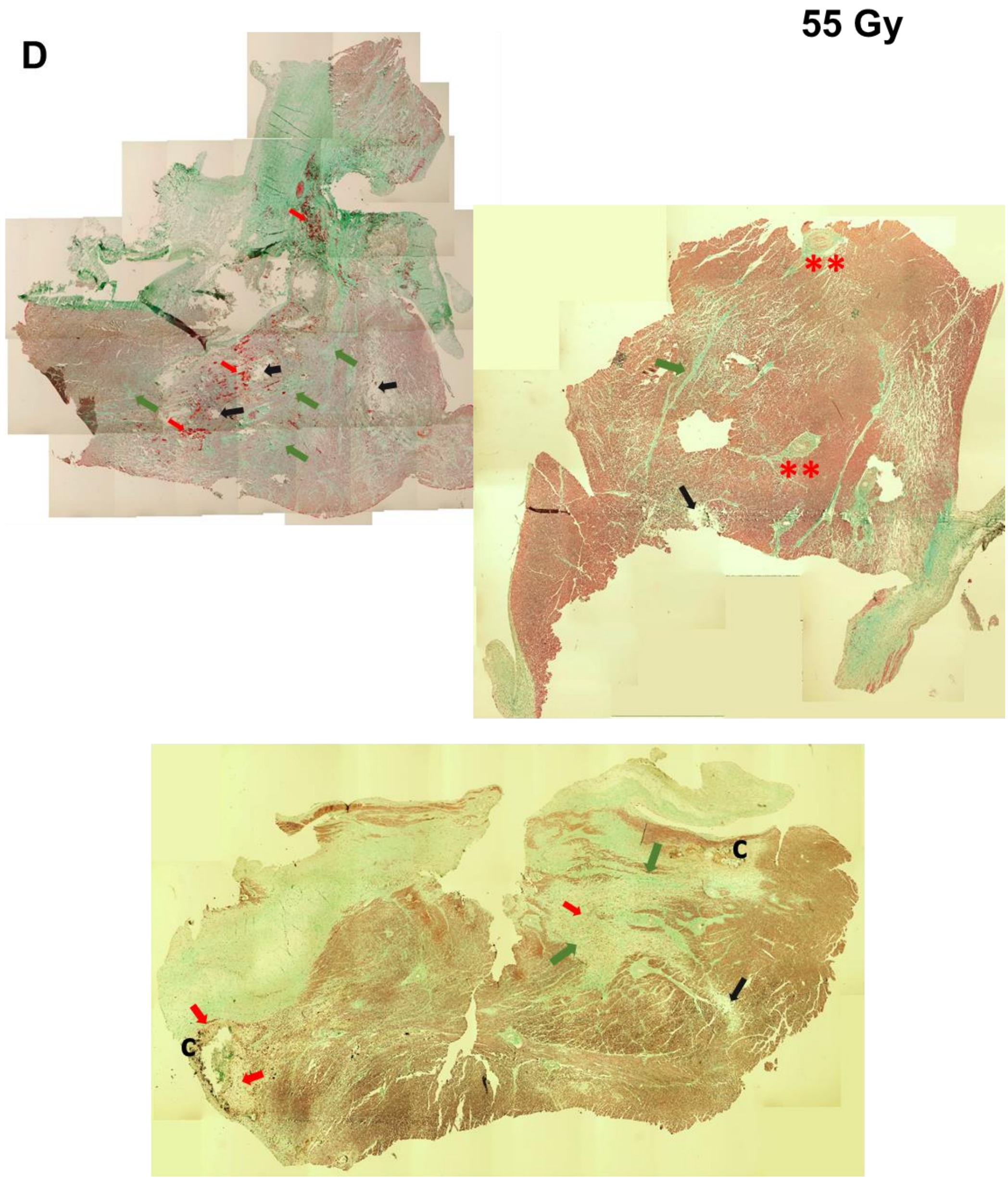

Fig. S5: Overview of fibrosis induction in the atrioventricular junction (AVJ) ablation of pig hearts 6 months after targeted irradiation Carbon ions. Sections were stained with Masson-Trichrome, single pictures with 4x magnification were taken and stitched to create a merged overview picture of the whole section. In sham irradiated tissue (A), healthy myocardium can be found along with physiological connective tissue (e.g. of the cardiac skeleton) and normal vessels (*). In samples irradiated with 25 Gy (C), myocardium is almost unchanged. After irradiation with 40 or 55 Gy, interstitial collagen deposition increases (green arrows), bleeding into tissue is present (red arrows) and necrotic areas develop (black arrows) (C-D). Occluded vessels (**) as well as calcified areas (c) are present.
